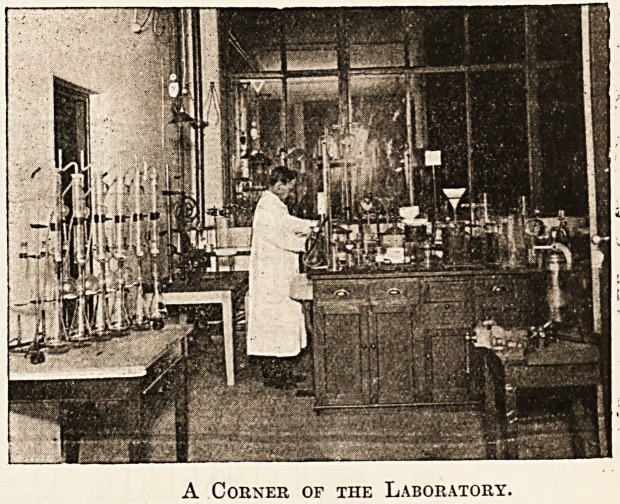# The Cancer Hospital, Fulham

**Published:** 1911-06-03

**Authors:** 


### THE CANCER HOSPITAL, FULHAM.

### THE NEW RESEARCH BLOCK.

To the brief account of the visit of the Duke and Duchess of Connaught to the Cancer Hospital, which we published last week, we are now at liberty to add the following further details. The steps leading to the main entrance were lined with nurses in their neat blue and white striped uniforms, with, at the head, the matron, Miss Sherratt, dressed in blue, and wearing the graceful veil of the army nursing service. Afterwards their Royal Highnesses visited the "Marsden" Ward, named from the founder of the hospital, Dr.

Cancer Hospital (The Research Building).

Marsden, where the patients are women, mostly serious cases, who find there a life-long refuge. The following is the official description of the research block, which has been planned in accordance with the requirements of the scheme of research adopted by the hospital, and provides for study in every branch of medical science bearing on the subject of cancer. The main entrance on the ground floor leads to a spacious hall, around which are disposed the various offices of administration: The Director's room, adjoining which is his laboratory, the office and attendant's room, and a large reference and reading room.

In the basement there is a machine room, installed with machinery and electrical plant, adjoining it a large cold store and refrigerator, and leading off the machine room is a centrifuge room. On this floor are also placed the laboratory for experimental electro-radio-physics, and a large dark room and photographic department.

A special laboratory has been set aside on this floor for preparatory chemistry which is equipped with mills, presses, combustion furnace, and an apparatus for the preparation of distilled water. There is also a room for sterilization and preparation of culture media, an underground laboratory for working at an equable temperature, and workshops, stores and other offices.

A Corner of the Laboratory.

The upper part of the building is mainly devoted to the Research laboratories which are ten in number; half are set aside for the important branches of chemistry and physics. The same arrangement has been maintained as in the lower part; the laboratories, which for the most part inter-communicate, also open into the central hall.

The institute is lighted throughout with electricity. It is also thoroughly equipped with gas and hot and cold water, and is heated by radiators.

## Figures and Tables

**Figure f1:**
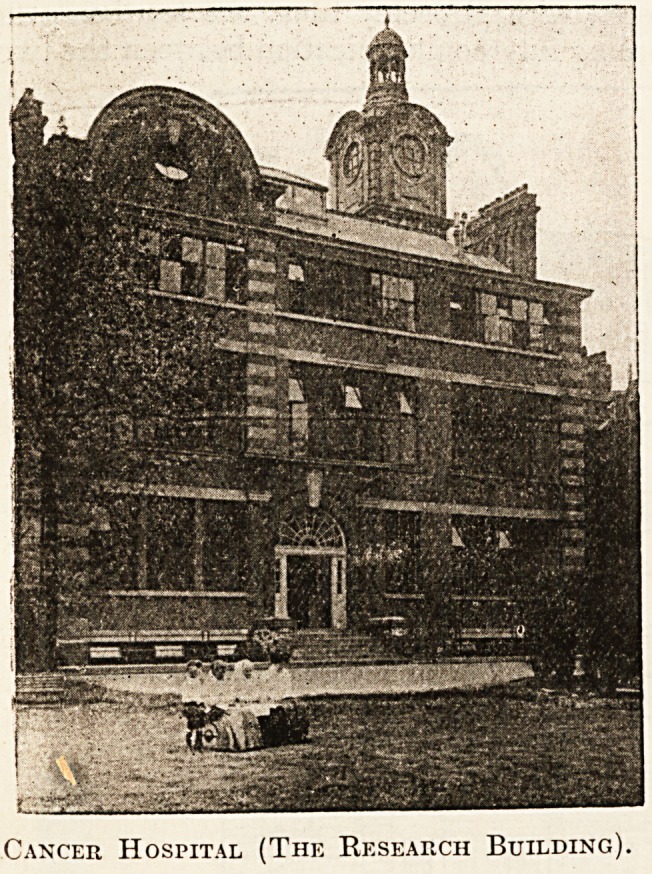


**Figure f2:**